# Comprehensive Analysis of Retracted Publications in Dentistry: A 23-Year Review

**DOI:** 10.1155/2020/8881352

**Published:** 2020-12-27

**Authors:** Shannon Samuel, Joe Mathew Cherian, Abi M. Thomas

**Affiliations:** Department of Pedodontics and Preventive Dentistry, Christian Dental College, Ludhiana 141008, Punjab, India

## Abstract

**Background:**

In the modern tech-savvy era, scientific literature publication remains the optimal way to disperse knowledge, even if it has transformed from print to mostly electronic. With the new and improved publication methods, also come more scrutiny and analytic criticism of the scientific work. It becomes even more important in this context to rectify flawed scientific work responsibly. This present study was undertaken to help clarify the process and causes of retractions occurring in the dental community and analyse its reasons. *Methodology*. A total of 8092 PubMed indexed articles were scanned from the online libraries, and individually scanning for author details, place of study, subspecialty of research, funding, dates of original publication, and retraction notices issued along with journal specifics such as type and impact factors, country of publishing was compiled and analysed by two authors. The dataset was then collaboratively analysed using Panda's Library in Python software as an analysis tool for data preparation and for frequency analysis. The estimates were presented as mean differences (MD) and 95% confidence intervals (95% CI).

**Results:**

The present study had a compiled dataset of 198 articles after screening and revealed that maximum retractions of dentistry-related research originated from India (25.3%) and, on average, took 2.6 years to be issued a retraction notice. We also deciphered that the USA retracted maximum dental articles (34.8%), and plagiarism was cited as the most common (38.02%) reason for doing so. The present study also brought to light that there was a trend for lower impact factor-dental journals in retracting maximum articles, most of which were nonfunded (62.16%). The results signify that 63.78% of all retracted papers continued to be cited postretractions.

**Conclusions:**

The retractions happening in the field of dental literature are currently too time-consuming and often unclear to the readers. The authors would like to conclude that the retracted papers were mostly from India and Spain mostly related to endodontics or prosthodontic research. All of this warrants the need for better scrutiny and reforms in the area.

## 1. Introduction

The publication of a scientific work remains a ring of fire every researcher must pass through which is held in place by the age-old commandment of “publish or perish.” Dentistry is no different. The publication of contemporary research and practices guides us towards improved more “evidence-based” treatment methods, learning, patient management, and teaching. Retraction of an inaccurate work is one of the means to ensure self-correction for any academic community [1]. Although correction of the scientific record is laudable per se, an inaccurate or fraudulent research can cause huge harm, diverting other scientists to unproductive lines of investigation, leading to the unfair distribution of scientific resources, leading to erroneous patient care [2]. Past years have seen a steady rise in the number of retracted publications in the biomedical community. It is not clear if the increase in the number of retractions is a result of an increase in the rate of incorrect research in toto or an upsurge in the rate at which flawed articles are recognized and withdrawn [3].

As of March 2020, there are around 139 dental journals listed in PubMed [4]. These publish hundreds of articles in a year alone. Overall, the number of journals that report retractions have also escalated. In 1997, just 44 journals testified retracting a paper. By 2016, that number had grown more than 10-fold to 488 [5]. Thus, the understanding of retracted articles is a necessary tool to wield to prevent flawed and improper research and data analysis. In the past, a few grave instances of scientific misconduct have gained notoriety in the dental community, but it failed to create the impact it should have, hinting at ignorance or even tolerance [6]. There are guidelines in place to ensure ethical research and publication, issued periodically by Committee of Publication Ethics (COPE) [7], National Library of Medicine [8], and the International Committee of Medical Journal Editors [9]. Failure to abide by these guidelines can lead to retraction of an article from the online or printed journals.

Retracted work can impact the students, authors, and scientific community negatively, if there is incomplete adherence to retraction guidelines. Even then, it is common to find unclear one-line retraction notices which do not explain or lack the justification of rationale for retraction [10, 11]. This creates a void in our understanding as dental researchers and practitioners about the boundaries of scientific misconduct which could warrant a retraction or withdrawal.

In recent years, there have been various articles studying the inclinations of retracted papers from different disciplines in the biomedical field most recently by Vuong et al. [12] where the boom in frequency of retraction in scientific publications and the necessity to learn from it was emphasized. There have been a few publications regarding retractions of dentistry-related scientific works as well [12]. Still, they all lacked the specificity regarding the pattern of retractions and the reasons leading to it.

Hence, the present study was undertaken to comprehensively analyse recent trends of dentistry-related articles being retracted from “PubMed” along with understanding the reasons for retraction, time frame of each retracted article, type of journal, and studying the trends behind these retractions according to the demographics of the researchers and the place of publication.

## 2. Methodology

The present study did not involve any human interactive examination and thus did not require clearance by the Institutional Ethics Committee. All investigations and evaluations of search results were performed by two investigators conducting the research parallelly. For the present study, articles in the PubMed search engine (MEDLINE and PMC indexed journals) were included. The investigation was carried out online from the institute web server at Christian Dental College, Ludhiana, India.

The primary Medical Subject Heading (MeSh) used to identify articles was “retracted publications” that yielded 7735 results. A number of additional meSh terms searched subsequently were “retracted publications dentistry” (*n* = 146) and “withdrawn publication dentistry” (*n* = 32). Additionally, a search was made on the website http://www.retractionwatch.com in order to identify retractions. The search term used there was “dentistry” that yielded 179 results. Once all these publications were identified (total = 8092 publications), they were screened individually by two investigators to identify the papers satisfying the inclusion criteria.

Study selection/inclusion criteria
  - All retracted articles relating to dental, orofacial, or craniofacial structures
  - All retracted articles relating to dental clinical or laboratory settings
  - All retracted articles relating to any dentistry-related case reports
  - Any retracted dental research based on animal studies
The investigators excluded the publications that
  - Were repeated in the search (*n* = 83)
  - Were not related to dentistry (*n* = 7981)
  - Were not a part of PubMed database (*n* = 12)
  - Were only partially retracted (*n* = 2)
  - Were only mentioned as “corrections” (*n* = 2)
  - Were a part of results due to MeSh terms (*n* = 12)

All the articles were evaluated by two investigators independently reviewing the titles and abstracts against the inclusion criteria for potentially eligible publications. Discrepancies were resolved with consensus by a third author.

The study was performed in 3 steps:

- Collection of data
- Data analysis
- Tabulation and computation of results

### 2.1. Collection of Data

**Figure 1:**
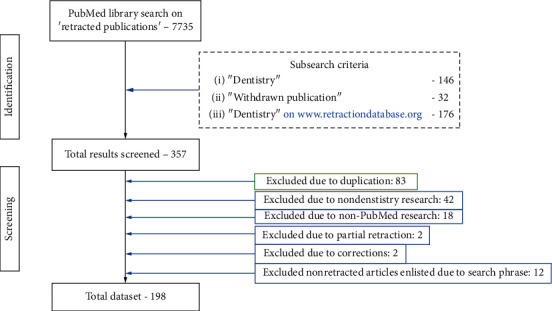
Flowchart depicting the steps in the methodology of identification and screening while assimilating the final database of articles. Final *n* = 198 articles.

The primary search was made by two authors independently on PubMed database for retracted articles in the field of dentistry from database creation to 29/07/2020. The first two steps comprised of identifying and screening of the results found as evident in Figure 1.

Third, both the investigators individually extracted data variables from each article.

(i) Author(s) name
(ii) Country of study
(iii) Branch relating to research
(iv) Funding received, if any
(v) Watermark present in the article retracted, if any
(vi) Citations received by the article after the retraction notice issued date, if any
Information regarding the dates and time frame
  - Date of original publication
  - Date of retraction notice publication
Information related to journal
  - Name of publishing journal
  - Scope of the journal—dental/medical or both
  - Impact factor of journal

Fourth, once the final dataset was compiled, we then searched for each corresponding retraction notice. Based on each notice, the retractions were categorised as per the Committee of Publication Ethics (COPE) guidelines [7]. The retractions were also scanned for the bidirectional link present between the original article and the retraction notice. Any reason not enlisted by COPE guidelines was specifically marked.

Fifth, the citations of each article were noted from the data provided by PubMed on the original page of the article under the heading “cited by.” For the articles retrieved from retractionwatch.com, these data were collected from accessing the original carrier journal website of the given article and checking the “metrics,” for example, PlumX for Elsevier journals and citation metrics for Wiley journals. For the purpose of this study, investigators only recorded the number of citations made after the issue of retraction notices. On instances where these data were not available or nonaccessible, a note was made.

Sixth, the information about the individual journals was collected from the SCImago search. Only the data available on the site was taken as authentic and the journals not indexed on SCImago were so marked. Finally, the authors collaboratively analysed the collected data. Any disputes were resolved by discussion with a third investigator.

### 2.2. Data Analysis

Statistical analysis of retracted articles was performed with respect to various extracted data variables: cause of retraction, place of study, time taken for retractions, field of study, journal specifics, author specifics, and if the research was funded. The distribution of retracted articles was analysed to isolate the features showing repetitive tendencies. Panda's Library in Python software version 3.7 was used as the analysis tool for data preparation and for frequency analysis. The features best describing the dataset which gave insight into the trends of retracted articles were identified. Based on the derived data frequency for the feature set, different histograms were plotted.

## 3. Results

Our current review compiled a dataset of 198 retracted papers between the years of 1998 and 2020 all relating to dental research.

After consideration of the data compiled, the authors tabulated in detail the countries of original research and the nation where the article was published (Table 1).

**Figure 2:**
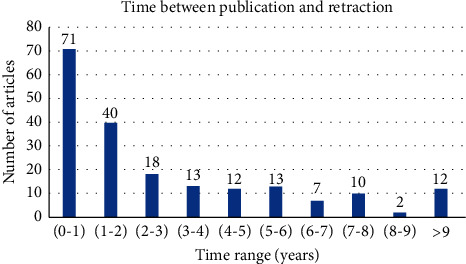
Graph showing the time duration between original paper publication and its retraction. *N* = 198.

The date of original paper publication and the date of retraction notice publication were analysed to calculate the average time lag between original publication and retraction of the article as charted in Figure 2. By the present estimation, we found that retractions in the past five years (2015–2020) have occurred 25% faster as compared to the previous decade (2005–2015).

**Figure 3:**
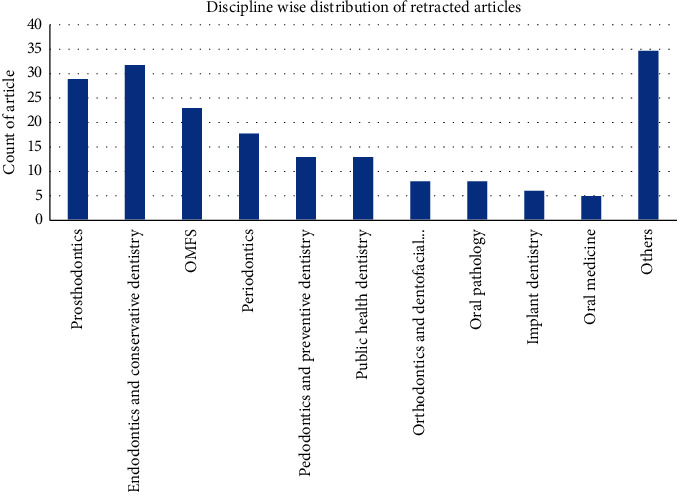
Graph showing branch-wise distribution of all dentistry-related retractions. The *x*-axis shows the discipline where the study was undertaken and *y*-axis the number of articles retracted.

Inspection of the dataset showed that majority of the publications were performed primarily from one or more dental departments (*n* = 133). Out of these, endodontics which amounted to 16.84% had most retractions followed by prosthodontics (15.26%) (Figure 3).

**Figure 4:**
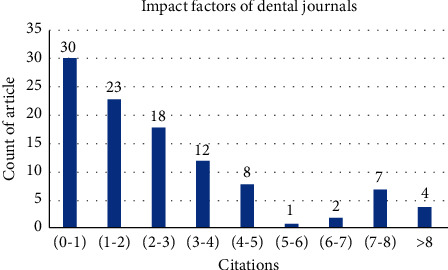
Graph depicting number of articles retracted from dental journals with impact factors within charted range. *N* = 89.

We recorded that out of the total, eighty-nine articles were published in dental journals, representing 44.94% of retracted publications since advent of the “retracted publication” index on PubMed. We deduced that majority of these publications were in peer-reviewed journals (98.9%). Impact factors of dental journals carrying these retracted publications are depicted in Figure 4.

**Figure 5:**
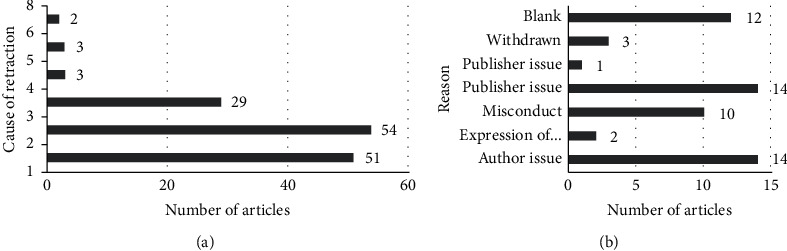
The above plotted graph shows the different enlisted causes of retraction (1–8) as per published COPE7 guidelines on the left and other reasons cited by some journals and their numbers on the right.

When we considered the reasons cited for retraction of the said papers, we found that most of the retractions were made according to COPE guidelines as shown in Figure 5.

By current assessment, 737 researchers are listed as authors of the 198 retracted papers included in the present review. Twenty-seven papers had a single author, whereas 149 studies had up to six authors and forty-nine publications had more than six authors. Nevertheless, some authors were found to be associated with multiple retracted articles. We were also able to assess that several authors (*n* = 41) received more than one retractions of their publications, out of which eleven authors had their work retracted more than twice until July 2020.

On further evaluation of each retracted article that we could find, we assessed that maximum retracted research was not funded (62.16%), and in thirty cases, no clear financial support was mentioned. Authors also deciphered that ninety-six of the total retracted articles in dentistry were watermarked as “RETRACTED” on the journal website clearly allowing for visual review. We were unable to access twenty-nine original articles after their withdrawal/retraction as the official journal website no longer carried them. It was calculated that 62% articles continued to be cited actively even after they had been issued retraction notices. We were unable to find data about twenty-one publications regarding citations and metrics.

## 4. Discussion

Whilst working on the present research, we are aware that there are varied terms that can be applied to refer the retraction of a paper like “retired,” “cancelled,” “self-retraction,” or “removal” [13]. In the present prevue, we have not made a distinction for any of the above.

### 4.1. Demographics

On analysing the patterns, we deduced that the maximum retracted papers within the dental community were authored or researched in India (25.3%) followed by Spain and USA as charted in Table 1. It has been suggested that nations producing more publications at a faster rate must simultaneously deal with a higher number of retraction notices [14]. Previous research studies, albeit in other discipline, signify that China was the nation producing maximum publication misconduct, followed by Iran and India [14, 15]. In our study, Asia ranks as the leader in this area (51.89%) which could be attributed to the want of resources at the academic institute level which is prefunctionary to system failure due to the strict hierarchy [16].

### 4.2. Time Period

Our study found that the average time lag of retracting an article was 2.6 years, although this time frame ranged from few months to about fourteen years as plotted in Figure 2. This average is in accordance with a study by Damineni RS et al. (2015) who observed a mean time of 2.8 years in 2012, compared to 2.2 years in 2013 [17]. Another study estimated that the first year of publication accounts for maximum retraction, with most of them being retracted in the first couple of years [12].

### 4.3. Discipline

Since we evaluated the retractions of scientific literature pertaining to dentistry, it made sense that majority of the retracted work originated by researchers affiliated with one or more dental disciplines (71.89%). Within, dentistry endodontics and prosthodontics amounted to the maximum retractions of literature (Figure 3). It is tough to comment on this trend as it could be attributed to the fact that primary authors of the retracted papers are affiliated with more than one discipline and the fact that different countries and universities define dental departments and disciplines uniquely. It is not always clear to classify the correct dental discipline involved due to blurred lines of an interdisciplinary research.

### 4.4. Journal Specifics

A journal's impact factor is the amalgamation of the number of citations in the present year to items published in the preceding two years and the number of substantive articles and reviews published in the same two years [18]. As per the current review, 89.44% of retractions were made by journals with impact factors lower than four as shown in Figure 4. A positive association between the higher impact factor and better research reporting has been previously reported by Peron et al. and Hua et al. 2015 [19, 20]. It is possible that a superior inspection is being imposed on publications in high impact journals and their headlighting the cutting-edge research can be the reason behind this trend [11]. On further analysis, it is evident that journals with impact factors <1 retracted majority of papers (28.8%). This could be the outcome of several reasons ranging from low level of scrutiny, lower standards of review, or flawed peer review in lower-ranking publications. Journals with high impact factors have taken the lead in policing their papers after publication. Two-thirds of 147 high-impact journals, most of them biomedical titles, have adopted COPE or similar policies to scrutinise the work they publish [20].

### 4.5. Cause of Retraction

The majority of the cause of retraction cited by various journals (95.67%) abided by COPE guidelines. Of these, we found the most common reason cited as plagiarism, which amounted for 38.57% followed by unreliable data and duplicate publications, as exhibited in Figure 5. Also, it should be understood that sometimes a paper can be retracted due to multiple cause (*n* = 4), as per our computations [1]. Our findings coincided with those reported by Grieneisen and Zhang [21] who listed the top three reasons for retractions as misconduct, primarily plagiarism, and author-initiated duplicate publication. The modern times has brought with it more digital survey and increased use of systematic reviews, which makes plagiarism easier to spot [22]. There can be the genuine confusion as one study could yield multiple outcomes impacting multiple areas of research. Moreover, the editors do not seem to have a common view on publication of papers with prior publication in abstract form in conference proceedings [22]. Apart from the COPE issued reasons, articles were also retracted as per our study due to a myriad of other reasons such as author issues and publisher issues as presented in Figure 5, the detailed analysis of which was beyond the scope of this research.

### 4.6. Postretraction

It is often seen that the retraction notices are abstruse, creating a section of such notices that do not give enough information regarding the cause of retraction [23]. We noticed eleven papers where no reason for retraction was cited and further fifty-three papers where the reason cited was vague or undiscernible. It is our understanding that sometimes authors are included in deciding the wording of a retraction notice, but it seems like this is a practice that should not be encouraged as it can be misleading and defeat the transparent nature of the whole transaction [2]. It will be well advised for all researchers to thus check the status of their referenced articles prior to publication [13].

Funded articles accounted for 25.9% of the current dataset. It can be assumed that fewer checks and offsets exist when funding is absent or from an internal source which can allow a wider latitude for misconduct or fabrication. It has been documented that research with external funding is quite rarely retracted. This is thought to be due to fewer checks imposed on internal or nonfunded research giving more latitude for authors to fabricate results [24].

Once retracted, it is important for the paper to be visibly marked. This visible watermark makes it easier for any reader to assess the authenticity of the paper and at the same time prevent the dissipation of invalid and incorrect data or research even by mistake. By the present review, we could find 63.78% of the papers that continued to be cited after their retractions. Now, the Committee on Publication Ethics, International Committee of Medical Journal Editors, and the National Library of Medicine advise that retracted publications continue to be available to the public, [7–9] but nonwatermarked versions of the article could be identified on 34.05% of all papers that were scanned in the present study dataset. Such practices greatly hinder the visibility and clarity to readers regarding the validity of that particular literature [13].

All of this urges the scientific community as a whole and dental community specifically to bring about changes and alterations from within and from the authors, readers, and the publishers.

### 4.7. Limitations

In this article, postretraction citation was counted as those occurring immediately after the retraction notice. It is different from other research studies on the postretraction citation. Kim et al. [25] define the postretraction citation as “those occurring at least one year after the retraction, considering the index time for the notice of retraction and the time to publication after submission.” Therefore, this study's results may be different if the definition of the postretraction citation was that of Kim et al. [25].

## 5. Suggestions

### 5.1. For the Readers

We urge all who consume scientific publications via electronic media to pay attention to the bidirectional electronic link often accompanying a withdrawn/retracted paper. For instance, the reader should be alert towards literature indexes, such as Web of Science and Scopus, which link the original paper to retraction notice. Academic search engines (e.g., Google Scholar) do not as of now have any apparatus to detect retracted publications. Hence, the judgement falls on the reader to assess the validity of any published literature [21].

### 5.2. For the Authors

Enforcing Retraction Guidelines (RG) and the Enhancing Quality and Transparency of health Research (EQUATOR Network) guidelines and their strict adherence will help all authors in better dental research conduct [20]. It will also be in the interest of all investigators to familiarize themselves with the aforementioned committees' ethical guidelines.

### 5.3. For the Publishers

We would like to urge all the editors to adopt a steady guideline while tackling published work retraction [26]. Also, it would be wise that all citations made postretraction/withdrawal of any article be intimidated to the publishers. More awareness can be generated regarding ethical publishing and research by circulating information about research guidelines through digital means [20]. Making the retraction notices visible, clear, and comprehensible will achieve the retraction's purpose and will also be beneficial for all those who come across it. CrossMark and FundRef are services offered that could aid in this venture to provide the most updated information regarding the article retraction status and funding information [24].

1. Along with software to identify and flag plagiarism, authors must also be asked to submit “raw data” which can be analysed by the editorial board to prevent any misconduct or data manipulation [27]
2. Obligatory training of the dental students in both undergraduate and postgraduate programs in relation to academic integrity is required within curriculum and institutions' involvement in this process could be instrumental
3. Guidelines on ethical editing and for retracting articles issued by COPE are a huge aid in enacting the ethical conduct of research and preventing fraud, but we found that even these guidelines need to be more inclusive and revised periodically in order to be up-to-date as gatekeepers of ethical dental publishing

## 6. Conclusion

The authors would like to conclude that the process of retraction or withdrawal of flawed dentistry-related publication is time-consuming and often opaque. The above research brings to light a number of areas like the fact that retractions are more common in nations such as India, Spain, and the USA that have a higher research potential and should be taken as a positive indicator. The need for scrutiny and careful ethical work also is encouraged via the suggestions made by the authors.

## Figures and Tables

**Table 1:** The country-wise distribution of the place of study and the count of article.

Place of study	Count of article
India	50
Spain	21
USA	20
Japan	14
P.R. China	13
Brazil	10
Saudi Arabia	9
UK	9
Iran	7
Italy	6
Others	39

In the present study, majority of retracted publications were originally from India (25.3%) followed by Spain (10.6%) and the USA (10.1%).

## Data Availability

The article data used to support the findings of this study are freely available on the domain https://pubmed.ncbi.nlm.nih.gov/. This information was freely accessed and the supplementary files of extracted data can be requested from the corresponding author.
